# Apatinib exhibits synergistic effect with pyrotinib and reverses acquired pyrotinib resistance in HER2-positive gastric cancer via stem cell factor/c-kit signaling and its downstream pathways

**DOI:** 10.1007/s10120-020-01126-9

**Published:** 2020-10-08

**Authors:** Beibei Su, Tingting Huang, Yu Jin, Han Yin, Hong Qiu, Xianglin Yuan

**Affiliations:** Department of Oncology, Tongji Hospital, Tongji Medical College, Huazhong University of Science and Technology, 1095 Jiefang Avenue, Wuhan, 430030 Hubei China

**Keywords:** Pyrotinib, Gastric cancer, HER2, Apatinib, Pyrotinib resistance

## Abstract

**Background:**

Recently, progress has been made in the development of targeted therapies for human epidermal growth factor receptor 2 (HER2)-positive gastric cancer (GC). However, drug resistance has severely limited the efficacy of anti-HER2 therapies. Pyrotinib is a novel pan-HER inhibitor. Although it is effective in HER2-positive GC treatment, its efficacy in combination with apatinib and associated resistance mechanisms in HER2-positive GC remains unclear.

**Methods:**

In this study, the combination effects of pyrotinib and apatinib were examined in two pyrotinib-sensitive GC cells and xenografts. The RNA sequencing was used to determine the underlying mechanisms of acquired pyrotinib resistance. The role of imatinib and apatinib in reversing pyrotinib resistance was tested in pyrotinib-resistant cells and xenografts.

**Results:**

Here, we reported that a combination of pyrotinib and apatinib exhibits synergistic effect in HER2-positive NCI-N87 xenografts, and showed enhanced antitumor efficacy in HER2-positive GC, both in vitro and in vivo. Moreover, up-regulation of the stem cell factor (SCF) levels, and the PI3K/AKT and MAPK pathways was associated with acquired pyrotinib resistance in HER2-positive GC. Mechanistically, we demonstrated that the activation of the SCF/c-kit signaling and its downstream PI3K/AKT and MAPK pathways mediated pyrotinib resistance by promoting cell survival and proliferation. Imatinib and apatinib augmented the sensitivity of pyrotinib-resistant cells and xenografts to pyrotinib, by blocking SCF/c-kit signaling.

**Conclusion:**

These results highlight the effectiveness of pyrotinib combined with apatinib in HER2-positive GC and acquired pyrotinib resistance, thus providing a theoretical basis for new treatment methods.

**Electronic supplementary material:**

The online version of this article (10.1007/s10120-020-01126-9) contains supplementary material, which is available to authorized users.

## Introduction

Rapid progress in genomic analysis and sequencing technology has opened doors to a new era of precision treatment, and targeted therapy has become one of the most important treatment regimens for cancer. Gastric cancer (GC) ranks third among the leading causes of cancer deaths worldwide; however, targeted therapies for GC are very limited [1]. Although clinical trials for GC treatment have continued to emerge, most of them have failed [2]. Among the molecular targets studied, human epidermal growth factor receptor 2 (HER2) is the most widely used, and its clinical significance has been clearly described. According to reports, about ~ 12% to ~ 20% of GC patients are HER2-positive, with rates in the adenocarcinoma of esophagogastric junction (AEG) as high as 32.2%, indicating the requirement for an effective treatment strategy for HER2-positive GC [3, 4].

Trastuzumab is considered a first-line treatment for HER2-positive advanced gastric cancer (AGC); however, the objective response rate (ORR) is reportedly only 47% [5]. Moreover, trastuzumab cardiotoxicity remains an issue, and trastuzumab resistance is an unavoidable problem, given the lack of a standard treatment for trastuzumab-resistant HER2-positive AGC. Previous studies show that a critical drug-resistance mechanism of trastuzumab is the compensatory signal transduction of other HER receptors [6, 7]. Therefore, this suggests that the pan-HER inhibitor pyrotinib might be an effective treatment strategy for HER2-positive AGC.

Pyrotinib is a novel oral, potent, and irreversible pan-HER tyrosine kinase inhibitor (TKI), which inhibits the epidermal growth factor receptor (EGFR)/HER1, HER2 and HER4. Preclinical studies have revealed the excellent antitumor activity of pyrotinib both in vitro and in vivo in HER2-positive advanced solid tumors [8, 9]. In a phase II randomized controlled trial (RCT) of pyrotinib in HER2-positive advanced breast cancer patients, a combination of pyrotinib and capecitabine significantly increased the ORR (78.5% vs. 57.1%) and extended progression-free survival (PFS) (18.1 vs. 7.0 months) as compared with combined lapatinib and capecitabine therapy. Moreover, the patients tolerated this treatment regimen well and benefitted from pyrotinib, irrespective of prior treatment with trastuzumab [10]. This trial motivated the acceptance of pyrotinib by the China Food and Drug Administration (CFDA) for advanced breast cancer treatment, and based on its remarkable antitumor efficacy in HER2-positive breast cancer patients, we evaluated its application for the treatment of HER2-positive GC in the present study.

Currently, a phase I trial of pyrotinib (NCT03480256) is underway in HER2-positive AGC patients [11]. Although no data have been announced, there have been previous clinical applications of pyrotinib in patients with HER2-positive AGC. Pyrotinib was reportedly effective in a HER2-positive AGC patient demonstrating resistance to trastuzumab and lapatinib [12], underscoring pyrotinib efficacy in patients showing trastuzumab resistance. Because GC is highly heterogeneous, the efficacy of pyrotinib alone is limited in HER2-positive AGC patients; therefore, combining pyrotinib with other targeted drugs might improve its utility.

Apatinib is a small-molecule TKI that selectively inhibits vascular endothelial growth factor receptor 2 (VEGFR2), and is also effective against Ret, c-kit, and c-src [13]. Apatinib reportedly exhibits a wide range of antitumor activity in advanced solid tumors [14, 15], and to date, it is the first small-molecule inhibitor approved for AGC treatment. Although no clinical trials have reported apatinib efficacy in HER2-positive AGC, studies reported partial response in a trastuzumab-resistant HER2-positive AGC patient that subsequently experienced 8 months of PFS following apatinib treatment [12]. These data suggest apatinib as a potential therapeutic option for HER2-positive AGC and trastuzumab resistance.

Both pyrotinib and apatinib are currently used independently for GC treatment; however, detailed studies on their combinatorial use for treating HER2-positive AGC have not been found. Therefore, we hypothesized that a combination of the two inhibitors would show a synergistic effect in HER2-positive AGC. Additionally, because drug resistance is a common limitation of targeted therapies, and resistance to anti-HER2 therapies has already been reported in HER2-positive AGC patients, understanding the mechanisms behind acquired pyrotinib resistance and identifying alternate treatment strategies are of great importance. Therefore, this study confirmed the role of combined application of pyrotinib and apatinib for the treatment of HER2-positive GC using in vitro and in vivo models. Furthermore, we established a pyrotinib-resistant cell line to examine the underlying mechanisms of acquired pyrotinib resistance, thereby laying the foundation for clinical research in HER2-positive AGC patients.

## Methods

### Cell lines and reagents

Human GC cell lines (NCI-N87, SGC7901, MGC-803, and MKN45) were purchased from the Cell Bank of Chinese Academy of Sciences (Shanghai, China). Human HER2-positive GC SNU216 cells were a generous gift from Professor Ruihua Xu, of Sun Yat-sen University Cancer Center (Guangzhou, China). NCI-N87-AR is a pyrotinib-resistant cell line obtained by continuous exposure of NCI-N87 cells to pyrotinib. All cells were cultured in RPMI-1640 (Hyclone, Provo, UT) containing 10% fetal bovine serum (FBS; Gibco, MD, USA) in an incubator with 5% CO_2_ and proper humidity, at 37 ℃. Pyrotinib (SHR1258) and apatinib (YN968D1) were obtained from Hengrui Medicine Co., Ltd (Lianyungang, China). Reagents were dissolved in dimethyl sulfoxide (DMSO) and stored at − 20 ℃. Recombinant human SCF was purchased from MedChemExpress LLC. (New Jersey, USA) and maintained at − 20 ℃ until further use.

### Cell viability assay

Cells (4000–8000 cells/100μL per well) were seeded in 96-well plates and incubated at 37 °C overnight. After 72 h of treatments, the Cell Counting Kit (CCK)-8 reagent (Promotor, Wuhan, China) was added at a concentration of 10%, and the absorbance of the samples was measured at 450 nm using a microplate reader (BioTek, VT, USA). The experiment was performed in triplicate and data were representative of three separate experiments.

### Colony formation assay

Cells were cultured in 6-well plates at a density of 800–1000 cells/well. The culture medium was renewed to normal after 72 h of different treatments and incubated for 2 weeks. The colonies were fixed and stained with 0.1% Crystal Violet, and colony (> 50 cells/colony) counts of the samples were then compared to that of the control group. The experiments were performed in triplicate and data were representative of three separate experiments.

### Transwell assay

Cells (5 × 10^4^) suspended in 200 µL serum-free medium were transferred into the upper chamber of a 24-well insert containing a membrane with an 8-μm pore size (Corning, New York, USA), and 500 μL medium containing 20% FBS was added to the bottom chamber. After incubation for 72 h, the cells that crossed the membrane were fixed and stained with 0.1% Crystal Violet for 30 min. For each sample, 10 fields of view were counted and the average was calculated (100 ×). The final data were obtained from three independent experiments.

### Cell apoptosis analysis by flow cytometry

5 × 10^5^ cells were obtained, washed twice with PBS, and re-suspended in 200 μL of 1 × binding buffer (BD Biosciences, NJ, USA), stained with 5 µL of FITC Annexin V, and incubated in the dark for 15 min, at 37 ℃. 5 µL of propidium iodide (PI) and 300 μL of 1 × binding buffer were then added 5 min prior to testing in the dark, at room temperature. Samples were measured by flow cytometry on an LSRFortessa cell analyzer (BD Biosciences), and the results were analyzed using FlowJo software (v.10.0; TreeStar, CA, USA). The final data were obtained from three independent experiments (Table 1).

**Table 1:** Synergism analysis in NCI-N87 xenograft models

	Apatinib		Pyrotinib		Combination		
Model	Dose range (mg/kg)	ED_50_^a^ (mg/kg)	Dose range (mg/kg)	ED_50_ (mg/kg)	Dose range (mg/kg, apatinib/pyrotinib)	ED_50_ (mg/kg)	CI^b^ (95% upper limit)
NCI-N87	1.5–150	31.19	0.2–20	3.95	1.5/0.2–150/20	4.54	0.26 (0.48)

^a^ ED_50_: median effective dose

^b^ CI: combination index

### RNA extraction and quantitative real-time PCR (qRT-PCR)

Total RNA was extracted using RNAiso Plus (Takara, Dalian, China), and reverse-transcribed to cDNA with the RevertAid first-strand cDNA synthesis kit (Thermo Fisher) according to a standard protocol. Real-Time qPCR was performed using the 7900HT Fast real-time PCR system (Thermo Fisher). *GAPDH* and *β-actin* were used as control and the 2^−ΔΔC^_t_ method was used to quantify the relative mRNA expression of the genes. The sequences of primers are enlisted in the Supplementary Table 2. The experiments were performed in triplicate and the data were obtained from three separate experiments.

### RNA sequencing and data analysis

RNA (1 μg) with an RNA Integrity number above 6.5 was used for following library preparation. Libraries with different indices were multiplexed and loaded onto an Illumina HiSeq instrument (Illumina, CA, USA) according to manufacturer’s instructions. The sequences were processed and analyzed by GENEWIZ (NJ, USA). A *P* value < 0.05 was used as the cut-off criterion.

### Protein extraction and western blot analysis

Total protein was extracted with a radioimmunoprecipitation assay (RIPA) buffer (Beyotime, Shanghai, China) supplemented with phenylmethylsulphonyl fluoride (PMSF) and phosphatase inhibitors (Servicebio, Wuhan, China). Protein quantification was then performed using the BCA reagent (Beyotime), following the manufacturer's instructions. The extracted proteins were loaded into 10% SDS-PAGE for separation, and transferred to a 0.45 μm polyvinylidene fluoride (PVDF) membrane (Millipore, Massachusetts, USA). Primary antibodies were added, and the membrane was incubated at 4 ℃ overnight. Subsequently, the membrane was incubated with secondary antibodies (Promotor) at room temperature for 1 h. Post incubation, the SuperSignal West Pico Chemiluminescent Substrate (Thermo Fisher) was added. The immuno-reactive bands obtained were analyzed using the G: BOX Chemi X system (Syngene, Cambridge, UK). The primary antibodies used are enlisted in the Supplementary Table 3.

### Xenograft models

Female nude mice (4–6-weeks old; BALB/c nu-nu) were purchased from the Hunan SJA Laboratory Animal Co., Ltd. (Changsha, China), and raised in a specific pathogen-free laboratory. Cells (5 × 10^6^ cells/100 μL) were injected subcutaneously into the left posterior side of each mouse, and mice were randomized into different groups (*n* = 6) when the tumors reached about ~ 100 mm^3^. The size of the tumor and the weight of the nude mice were measured every 3 days, and the tumor volume was calculated as follows: [(short diameter)^2^ × (long diameter)]/2. Mice were sacrificed after 21 days. Tumor samples were collected, photographed, and stained. The fixed dose ratio of apatinib/pyrotinib was determined from the median effective dose (ED_50_) of the two monotherapies to prevent bias. The combination index (CI) was calculated to determine the combined effect, with CI < 1, CI > 1, and 1 used to defined synergism, antagonism, and additivity, respectively. To determine the statistical relevance of CI < 1 [i.e., ln(CI) < 0], the variance of ln(CI) was calculated according to a previous described method [16]. All institutional and national guidelines for the care and use of laboratory animals were followed.

### Immunohistochemistry (IHC)

Immunohistochemistry was performed following the manufacturer’s instructions. In brief, tissue samples from xenograft models (*n* = 6 per group) were formalin-fixed overnight at 25 ℃, prior to embedding in paraffin. All tissue sections were obtained as slices from the paraffin blocks and the slices were incubated with primary antibodies against SCF (1:200, ab52603, Abcam, Cambridge, UK), Ki67 (1:200, #9027, Cell Signaling Technology, MA, USA), and CD31(1:2000, #3528, Cell Signaling Technology, MA, USA). The TdT-mediated dUTP nick-end labeling (TUNEL) assays were performed using a TUNEL in situ cell death detection kit (Sigma-Aldrich, MO, USA), according to manufacturer’s instructions. The results were then evaluated by two professional pathologists independently. The assays were performed by Biossci Biotechnology Co., Ltd. (Wuhan, China).

### Statistics

Chart generation and statistical calculations were performed using the GraphPad Prism software (v8.0; CA, USA), and SPSS (v19.0; NY, USA) was used for statistical analysis. All descriptive statistics were presented as the mean ± standard deviation (SD). Statistical comparisons between two experimental groups were performed using Student's *t* test. If multiple groups were compared, the one-way analysis of variance (ANOVA) was performed first. The least significant difference *t* test was applied if the overall difference was statistically significant. The main objective of statistical analysis in isobologram studies was to generate an upper confidence limit for the CI to determine whether the observed synergy was statistically relevant (CI < 1). A *P* < 0.05 was considered statistically significant for all tests.

## Results

### Combined treatment with pyrotinib and apatinib exhibits enhanced antitumor activity in HER2-positive GC cells in vitro

**Fig. 1:**
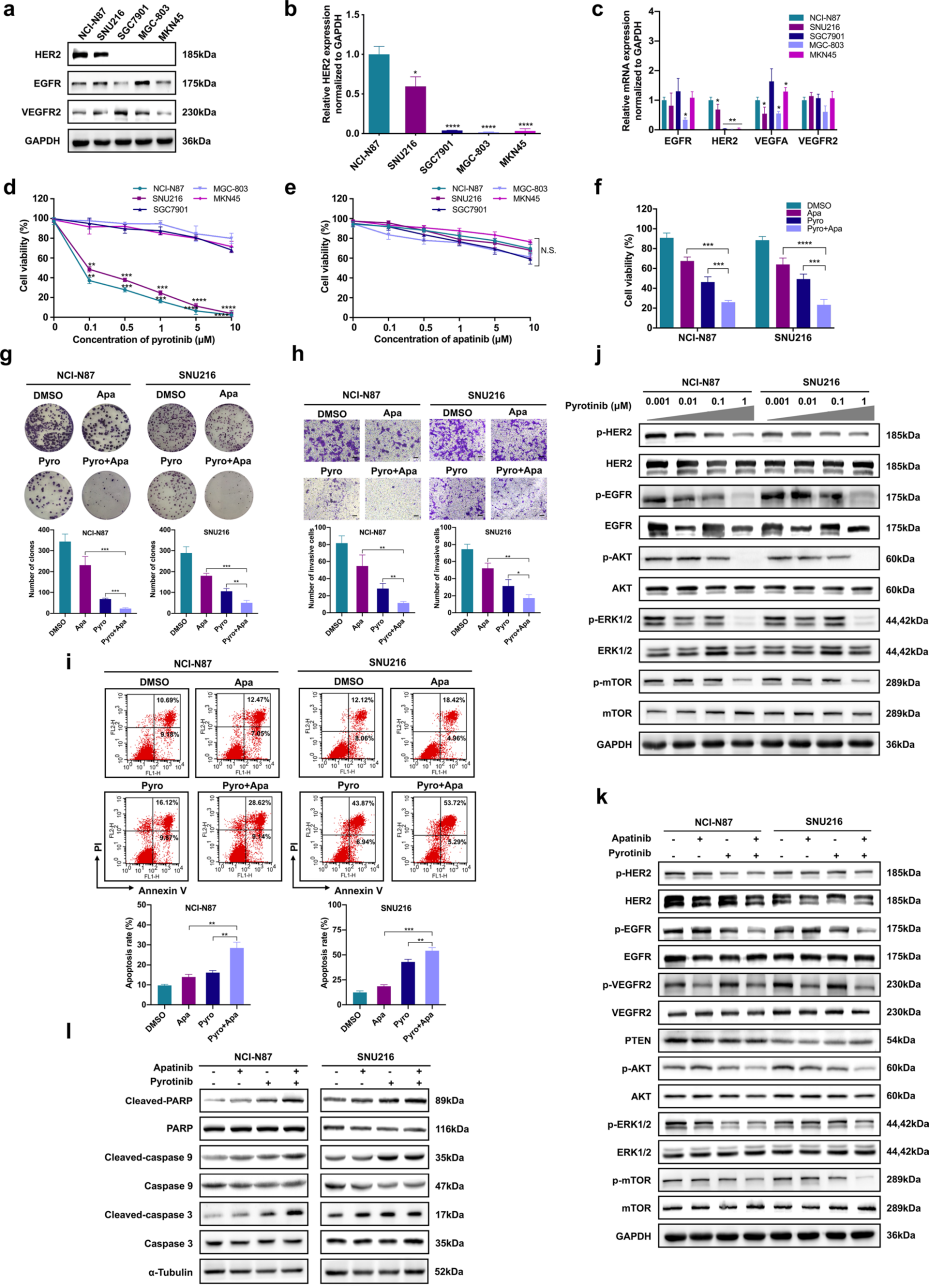
The combined treatment effects of pyrotinib (Pyro) and apatinib (Apa) in HER2-positive GC in vitro. **a** Protein levels of HER2, EGFR, and VGEFR2 in five human GC cell lines. **b** mRNA levels of *HER2* in five human GC cell lines. **c** mRNA levels of *EGFR, HER2, VEGFA* and *VGEFR2* in five human GC cell lines. **d, e** Cell viability of GC cell lines following pyrotinib and apatinib treatments via the CCK-8 assay. **f**–**i** NCI-N87 and SNU216 cells were treated with 10 μM apatinib, 0.1 μM pyrotinib or both for 72 h, followed by CCK-8, colony formation, and transwell assays and detection of cell apoptosis by flow cytometry. **j** NCI-N87 and SNU216 cells were treated with varying concentrations of pyrotinib for 24 h, followed by evaluation of molecules associated with EGFR/HER2 signaling by western blot. **k** NCI-N87 and SNU216 cells were treated with 10 μM apatinib, 0.1 μM pyrotinib or both for 24 h, followed by evaluation of molecules associated with EGFR/HER2 and VEGFR2 signaling by western blot. **l** Western blot assessment of levels of apoptosis-related proteins in NCI-N87 and SNU216 cells treated with 10 μM apatinib, 0.1 μM pyrotinib or both for 24 h. *P < 0.05; **P < 0.01; ***P < 0.001; ****P < 0.0001. *N.S.*, not significant

To establish suitable in vitro HER2-positive GC models, we first examined the expression of HER2 in five GC cell lines, and the NCI-N87 and SNU216 cells were revealed to be HER2-positive. The relative expression of EGFR and VEGFR2 receptors was also examined (Fig. 1a–c). We then evaluated the inhibitory effects of pyrotinib and apatinib on the five GC cells. Compared with the other cells, NCI-N87 and SNU216 cells were more sensitive to pyrotinib (*P* < 0.01) (Fig. 1d), whereas no significant difference was observed in the inhibitory activity of apatinib (Fig. 1e). The IC_50_ values of pyrotinib and apatinib for these GC cells are presented in the Supplementary Table 1. To examine the combination effect of pyrotinib and apatinib in HER2-positive GC, NCI-N87 and SNU216 cells were exposed to the two inhibitors, individually or in combination. The combination therapy significantly decreased the cell viability (*P* < 0.001), cell proliferation (*P* < 0.01) and invasion (*P* < 0.05), and increased cell apoptosis rate (*P* < 0.01) (Fig. 1f–i). To further investigate the associated mechanisms, the activity of the downstream pathways was evaluated. The EGFR/HER2 pathway was downregulated, in a dose-dependent manner, in NCI-N87 and SNU216 cells, on different pyrotinib treatments (Fig. 1j). The combination therapy downregulated the PI3K/AKT and ERK signaling pathways, and up-regulated levels of apoptosis-associated proteins in HER2-positive GC cell lines (Fig. 1k, l).

### Combined treatment with pyrotinib and apatinib exhibits enhanced antitumor activity and demonstrates synergy in HER2-positive GC xenograft models

**Fig. 2:**
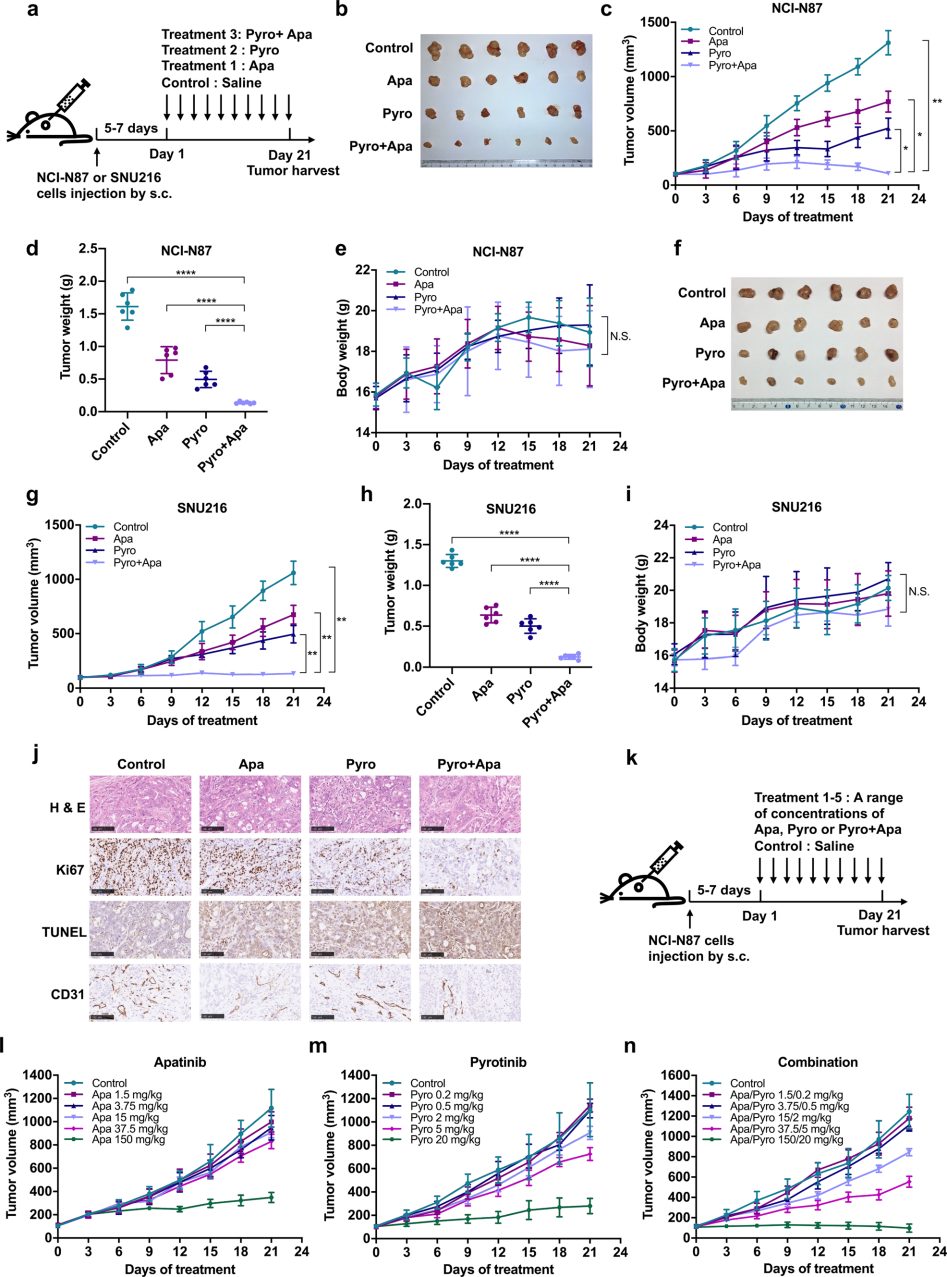
The antitumor efficacy of pyrotinib, apatinib and combined therapy in GC xenografts in vivo. **a** Schematic procedure of the in vivo experiments. Mice were subcutaneously (s.c.) injected with NCI-N87 or SNU216 cells. 5–7 days later, the mice were orally administered 75 mg/kg apatinib, 10 mg/kg pyrotinib or the combination daily for 21 days with saline administered as a control. **b, f** Representative images of NCI-N87 and SNU216 xenografts harvested after 21 days of treatments (*n* = 6). **c, g** Changes in tumor volume in mice. Data represent the mean ± SD. **d, h** Tumor tissue weight in each group on day 21. Data represent the mean ± SD. **e, i** Body weight gain/loss profiles of mice following treatments. Data represent the mean ± SD. **j** Representative images showing hematoxylin and eosin (HE) and IHC staining of xenograft tumor tissues. Scale bars, 100 μm. **k** Schematic procedure of the in vivo experiments to determine dose–response relationships for monotherapies and combined therapy. Mice were subcutaneously injected with NCI-N87 cells. 5–7 days later, the mice were orally administered a range of concentrations of apatinib (1.5–150 mg/kg), pyrotinib (0.2–20 mg/kg), or both (1.5/0.2–150/20 mg/kg) daily for 21 days with saline administered as a control. **l**–**n** Changes in tumor volume in NCI-N87 xenograft models. Data represent the mean ± SD (n = 6). *P < 0.05; **P < 0.01; ****P < 0.0001. *N.S.*, not significant

To evaluate the inhibitory effect of the combination treatment in vivo, the mice were inoculated with NCI-N87 or SNU216 cells and randomized into four groups treated daily with 10 mg/kg pyrotinib, 75 mg/kg apatinib, the combination of both inhibitors and saline as control (Fig. 2a). The combination group exhibited much stronger inhibitory effects in NCI-N87 (*P* < 0.05) and SNU216 (*P* < 0.01) xenografts compared to monotherapies (Fig. 2b–d, f–h). No significant difference in weight change was observed between the four groups (Fig. 2e, i). Tissues from the NCI-N87 xenografts were used for immunohistochemistry (IHC) analysis. Decreased Ki67 expression and increased cell apoptosis were observed in the combination group. No significant difference was observed in the expression of CD31 between the apatinib and the combination group (Fig. 2j). To examine the drug-specific interactions between pyrotinib and apatinib in HER2-positive GC, we assessed the tumor-suppressive efficacy of the inhibitors alone or combined in NCI-N87 xenografts (Fig. 2k–n). The ED_50_ of apatinib and pyrotinib was revealed to be 31.19 mg/kg and 3.95 mg/kg, respectively, and the combination group exhibited an ED_50_ of 4.54 mg/kg (pyrotinib: 0.54 mg/kg; and apatinib: 4.0 mg/kg). The CI was then estimated at 0.26, which indicated synergy between the two inhibitors (Table 1).

### SCF mediates acquired pyrotinib resistance through activation of the PI3K/AKT and MAPK signaling

**Fig. 3:**
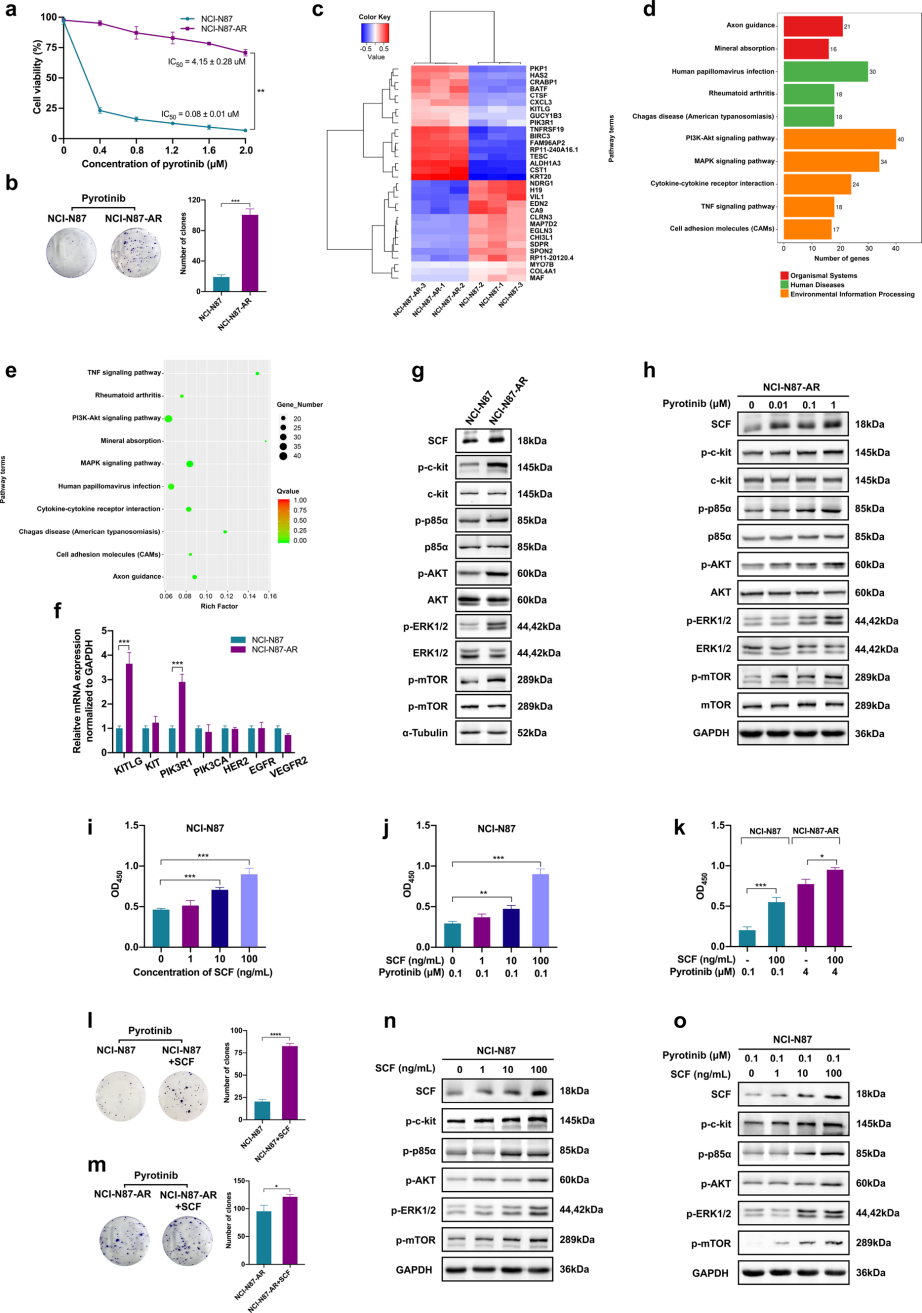
Analysis of different SCF levels between NCI-N87 and NCI-N87-AR cells and their effects. **a** Comparison of NCI-N87 and NCI-N87-AR cell viability by CCK-8 assay following pyrotinib treatments. Data represent the mean ± SD of triplicate experiments. **b** Colony formation assay of NCI-N87 and NCI-N87-AR cells treated with 1 μM pyrotinib. Data represent the mean ± SD. **c** RNA sequencing performed in NCI-N87 and NCI-N87-AR cells. The heat map shows the representative differentially expressed genes. **d, e** The top 10 enriched pathways according to Kyoto Encyclopedia of Genes and Genomes (KEGG) annotation classification. **f** qRT-PCR results for the indicated genes. Experiments were performed in triplicate. **g** Western blot analysis of proteins in the SCF/c-kit signaling pathway in NCI-N87 and NCI-N87-AR cells. **h** Western blot analysis of proteins in the SCF/c-kit signaling pathway in NCI-N87-AR cells treated with varying concentrations pyrotinib for 24 h. **i** NCI-N87 cells were treated with varying concentrations of SCF for 72 h, followed by determination of cell viability according to absorbance at 450 nm (OD_450_). **j** NCI-N87 cells were treated with 0.1 μΜ pyrotinib combined with varying concentrations of SCF for 72 h, followed by determination of cell viability according to OD_450_. **k** NCI-N87 and NCI-N87-AR cells were treated with 0.1 μM or 4 μM pyrotinib combined with or without 100 ng/mL SCF for 72 h, followed by determination of cell viability according to OD_450_. Data represent the mean ± SD of triplicate experiments. **l, m** Colony formation assay of NCI-N87 and NCI-N87-AR cells treated with pyrotinib (0.1 μΜ and 4 μΜ) combined with or without 100 ng/mL SCF. **n** Western blot analysis of proteins in the SCF/c-kit signaling pathway in NCI-N87 cells treated with varying concentrations of SCF for 72 h. **o** Western blot analysis of proteins in the SCF/c-kit signaling pathway in NCI-N87 cells treated with 0.1 μΜ pyrotinib combined with varying concentrations of SCF for 24 h. *P < 0.05; **P < 0.01; ***P < 0.001; ****P < 0.0001

To investigate the resistant mechanisms of pyrotinib in HER2-positive GC, a pyrotinib-resistant cell line, NCI-N87-AR, was constructed. The IC_50_ of pyrotinib in NCI-N87-AR cells was 4.15 ± 0.28 μΜ as compared with 0.08 ± 0.01 μΜ in NCI-N87 cells (*P* < 0.01) (Fig. 3a), and the colony number in NCI-N87-AR cells was significantly higher than that in NCI-N87 cells after pyrotinib treatment (*P* < 0.001) (Fig. 3b). Then, the RNA sequencing of the cells was performed. Compared to NCI-N87 cells, 736 genes were up-regulated and 795 genes were downregulated in NCI-N87-AR cells (*P* < 0.05 and > twofold change). The drug resistance-related genes *KITLG* and *PIK3R1* were among the most up-regulated genes, as shown in the heat map (Fig. 3c). The most enriched pathways revealed by the KEGG analysis were the PI3K/AKT and MAPK signaling pathways (Fig. 3d, e). The relative expressions of the genes were then verified by qRT-PCR (Fig. 3f). Comparing the expression of the *KITLG* and *PIK3RI* encoded protein (SCF and p85α subunit) and the related pathways, we found that the expression of SCF/c-kit and downstream PI3K/AKT and ERK pathways were significantly up-regulated in NCI-N87-AR cells relative to NCI-N87 cells (Fig. 3g). Furthermore, these pathways were up-regulated, in a dose-dependent manner, in NCI-N87-AR cells after different concentrations of pyrotinib treatment (Fig. 3h). No significant difference was found in the expression of HER2, EGFR, VEGFR2 (including their phosphorylation status) and PTEN levels between the two cell lines (Supplementary Fig. 1). To further determine whether the up-regulation of SCF played a role in pyrotinib resistance, we assessed the effects of SCF in NCI-N87 and NCI-N87-AR cells. As shown in Fig. 3i–k, the proliferation of both cells was significantly increased by SCF (*P* < 0.001 and *P* < 0.05), which neutralized pyrotinib-specific effects, with this finding subsequently confirmed by colony formation assays (Fig. 3l, m). Furthermore, on exposure to varying concentrations of SCF, with or without the addition of pyrotinib, the expression of SCF/c-kit, and downstream PI3K/AKT and MAPK pathways, were up-regulated in a dose-dependent manner (Fig. 3n, o).

### Imatinib enhances the sensitivity of NCI-N87-AR cells to pyrotinib by targeting c-kit and combined imatinib and pyrotinib treatment enhances antitumor effects in NCI-N87-AR xenografts in vivo

**Fig. 4:**
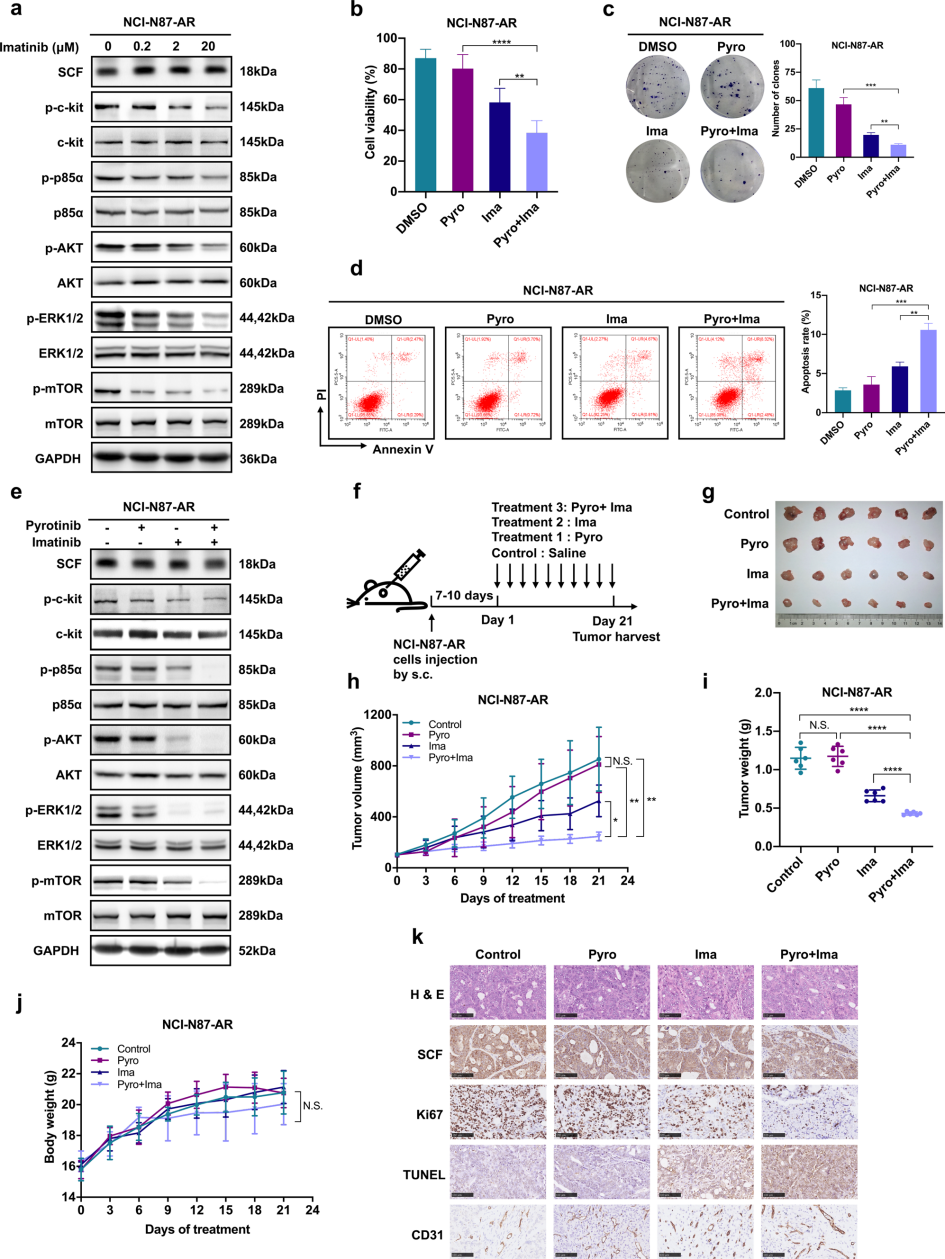
Imatinib (Ima) enhances the sensitivity of acquired pyrotinib-resistant HER2-positive GC to pyrotinib both in vitro and in vivo. **a** NCI-N87-AR cells were treated with varying concentrations of imatinib for 24 h, and proteins in the SCF/c-kit signaling pathway were assessed by western blot. **b**–**d** NCI-N87-AR cells were treated with 4 μM pyrotinib, 20 μM imatinib, or both for 72 h, followed by CCK-8 and colony formation assays and detection of cell apoptosis by flow cytometry. **e** Western blot performed to assess changes in protein levels associated with the SCF/c-kit signaling pathway in NCI-N87-AR cells treated with 4 μM pyrotinib, 20 μM imatinib, or both for 24 h. **f** Schematic procedure of the in vivo experiments. Mice were subcutaneously injected with NCI-N87-AR cells, and after 7–10 days orally administered 10 mg/kg pyrotinib, 100 mg/kg imatinib, or both daily for 21 days, with saline administered as a control. **g** Representative image of NCI-N87-AR xenografts harvested after 21 days of treatments (*n* = 6). **h** Changes in tumor volume in mice. Data represent the mean ± SD. **i** Tumor weight in each group on day 21. Data represent the mean ± SD. **j** Body weight gain/loss profiles of mice following treatment. Data represent the mean ± SD. **k** Representative images showing hematoxylin and eosin and IHC staining of xenograft tumor tissues. Scale bars, 100 μm. *P < 0.05, **P < 0.01, ***P < 0.001, ****P < 0.0001. *N.S.*, not significant

To verify the involvement of SCF/c-kit signaling pathways in mediating acquired pyrotinib resistance, we evaluated imatinib, a c-kit inhibitor, in acquired pyrotinib resistance in HER2-positive models both in vitro and in vivo [17, 18]. As shown in Fig. 4a, imatinib significantly inhibited SCF-induced c-kit phosphorylation and downstream molecules in the PI3K/AKT and ERK pathways in a dose-dependent manner. We then investigated the inhibitory effects of imatinib and pyrotinib, alone or in combination, in NCI-N87-AR cells. We found that combined therapy significantly decreased cell viability and proliferation, increased cell apoptosis, and downregulated PI3K/AKT and ERK signaling in NCI-N87-AR cells (Fig. 4b–e). To investigate whether combined therapy improves acquired pyrotinib resistance in vivo, mice were inoculated with NCI-N87-AR cells and randomized into four groups treated daily with 10 mg/kg pyrotinib, 100 mg/kg imatinib, the combination of both inhibitors, or saline as a control (Fig. 4f). The group receiving combined therapy exhibited stronger antitumor effects in NCI-N87-AR xenografts relative to those observed from monotherapy (Fig. 4g–i), with no significant difference in weight change observed between the four groups (Fig. 4j). IHC analysis of tissues from xenografts revealed similar SCF levels in the four groups, although decreased Ki67 levels and increased cell apoptosis were observed in the combined-treatment group, and no significant difference was observed in the CD31 levels between groups (Fig. 4k).

### Apatinib inhibits the activation of PI3K/AKT and ERK signaling by targeting c-kit, and enhances the sensitivity of NCI-N87-AR cells to pyrotinib

**Fig. 5:**
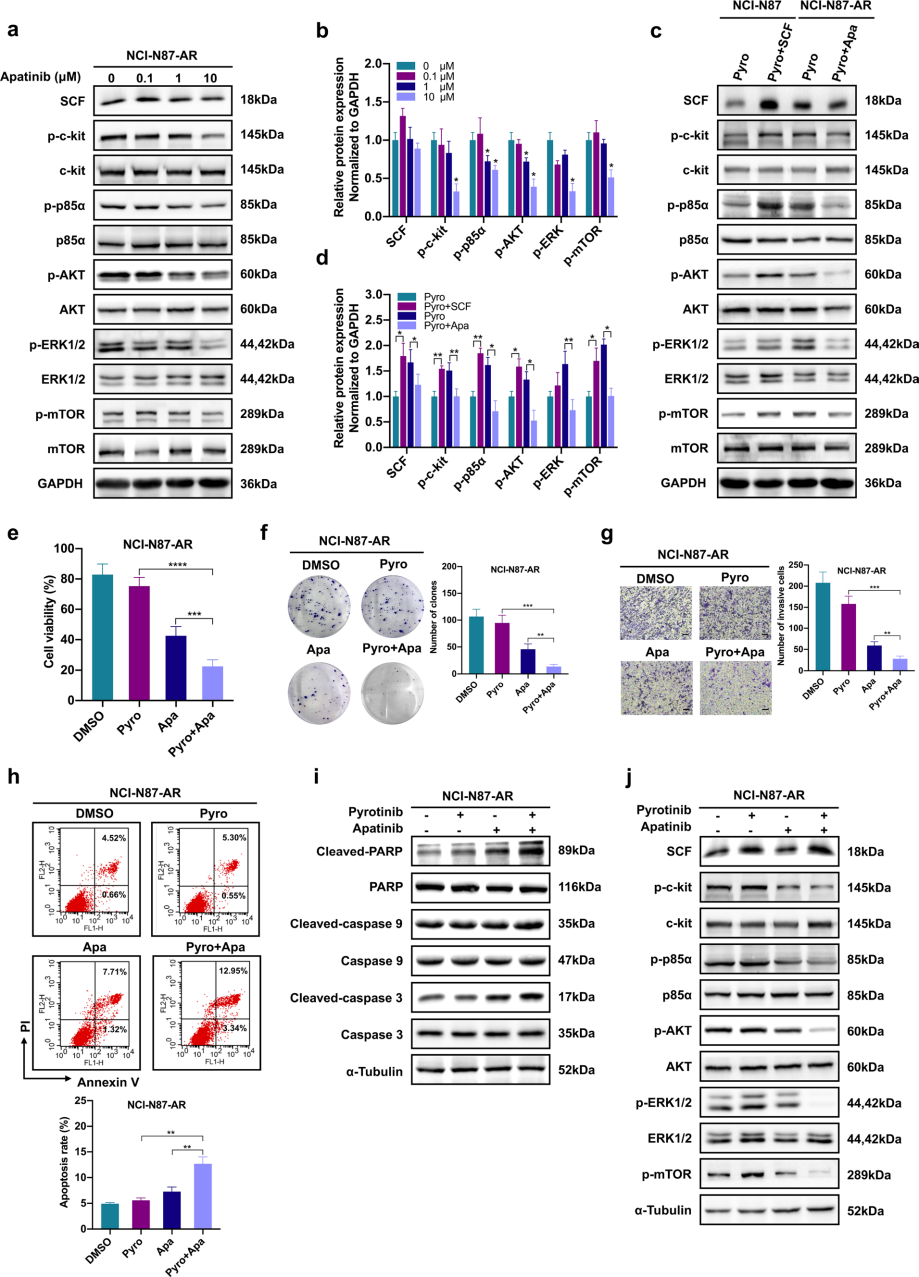
Apatinib enhances the sensitivity of NCI-N87-AR cells to pyrotinib in vitro. **a** NCI-N87-AR cells were treated with varying concentrations of apatinib for 24 h, followed by assessment of proteins in the SCF/c-kit signaling pathway by western blot. **b** Statistical analysis of SCF, p–c-kit, p-p85α, p-AKT, p-ERK, and p-mTOR levels in **a**. Data represent the mean ± SD. **c** NCI-N87 cells were treated with 0.1 μM pyrotinib combined with or without 100 ng/mL SCF and NCI-N87-AR cells were treated with 4 μM pyrotinib combined with or without 10 μM apatinib for 24 h, followed by assessment of proteins in the SCF/c-kit signaling pathway by western blot. **d** Statistical analysis of SCF, p–c-kit, p-p85α, p-AKT, p-ERK, and p-mTOR levels in **c**. Data represent the mean ± SD. **e**–**h** NCI-N87-AR cells were treated with 4 μM pyrotinib, 10 μM apatinib or both for 72 h, followed by CCK-8, colony formation, and transwell assays and detection of cell apoptosis by flow cytometry. **i** Western blot assessment of levels of apoptosis-related proteins in NCI-N87-AR cells treated with 4 μM pyrotinib, 10 μM apatinib or both for 24 h.** j** Western blot assessment of proteins in the SCF/c-kit signaling pathway in NCI-N87-AR cells treated with 4 μM pyrotinib, 10 μM apatinib or both for 24 h. *P < 0.05; **P < 0.01; ***P < 0.001; ****P < 0.0001

To investigate whether apatinib played a role in combating SCF-mediated pyrotinib resistance, the inhibitory effects of apatinib were examined in NCI-N87-AR cells. Apatinib significantly inhibited SCF-induced c-kit phosphorylation and the expression of downstream PI3K/AKT and ERK signaling in a dose-dependent manner (Fig. 5a, b), and enhanced the sensitivity of NCI-N87-AR cells to pyrotinib (Fig. 5c, d). Subsequently, we investigated the inhibitory effects of apatinib and pyrotinib, individually or in combination, in NCI-N87-AR cells, revealing that combined therapy significantly decreased the cell viability, proliferation and invasion, increased cell apoptosis (*P* < 0.01), as well as levels of apoptosis-associated proteins, and downregulated the PI3K/AKT and ERK signaling in NCI-N87-AR cells (Fig. 5e–j). The similar effects of apatinib also appeared in NCI-N87 and SNU216 cells (Supplementary Fig. 2, Fig. 1k).

### Combined treatment with apatinib and pyrotinib enhances the sensitivity of NCI-N87-AR xenografts to pyrotinib in vivo

**Fig. 6:**
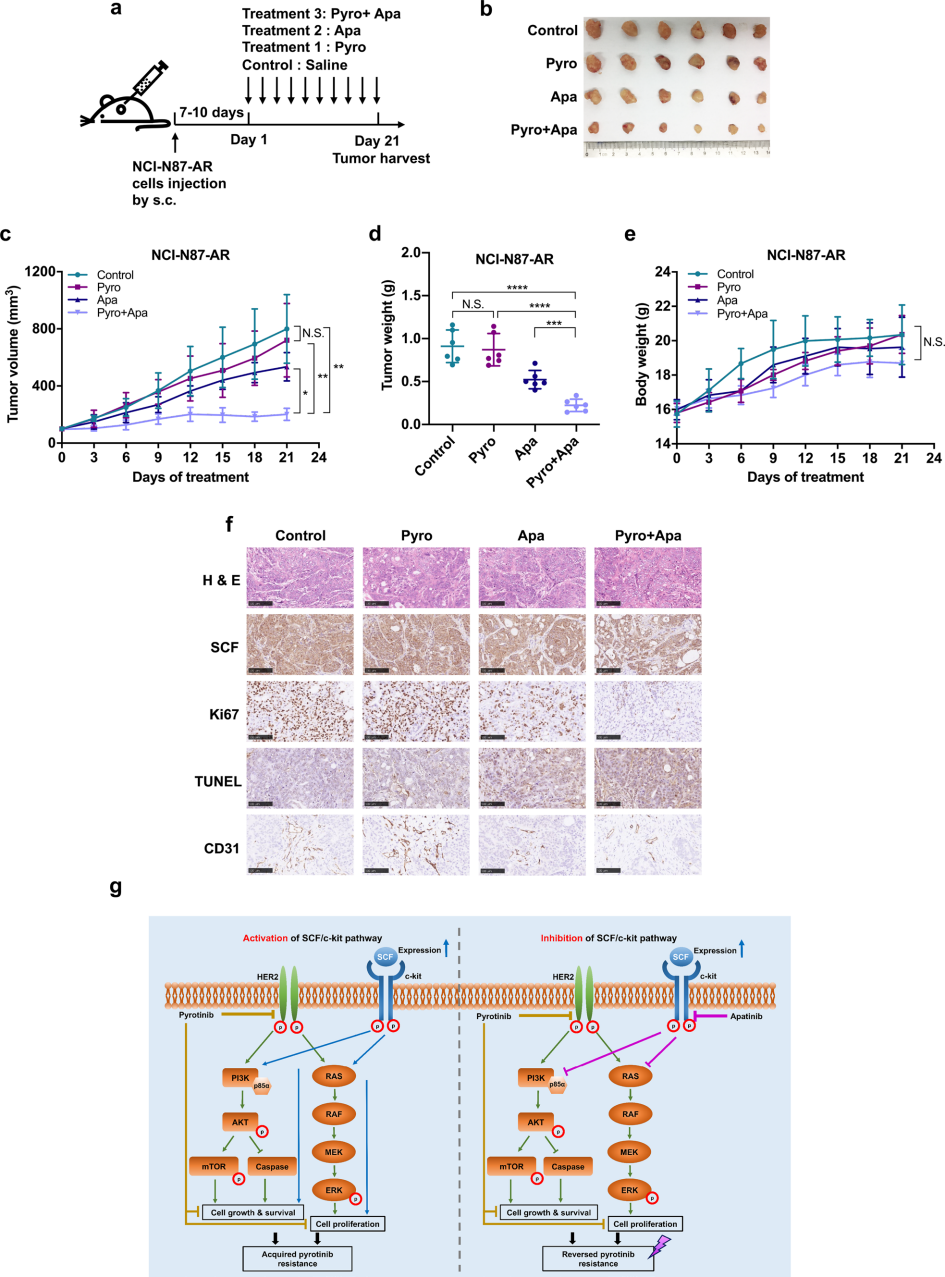
Apatinib enhances the sensitivity of NCI-N87-AR xenografts to pyrotinib in vivo. **a** Schematic procedure of the in vivo experiments. Mice were subcutaneously injected with NCI-N87-AR cells. 7 to 10 days later, the mice were orally administered 10 mg/kg pyrotinib, 75 mg/kg apatinib or both daily for 21 days with saline administered as a control. **b** Representative image of NCI-N87-AR xenografts harvested after 21 days of treatments (*n* = 6). **c** Changes in tumor volume in mice. Data represent the mean ± SD. **d** Tumor tissue weight in each group on day 21. Data represent the mean ± SD. **e** Body weight gain/loss profiles of mice following treatment. Data represent the mean ± SD. **f** Representative images showing HE and IHC staining of xenograft tumor tissues. Scale bars, 100 μm. **g** Proposed model illustrating the role of apatinib in overcoming acquired pyrotinib resistance of pyrotinib mediated by up-regulation of SCF in HER2-positive GC. Activation of the SCF/c-kit signaling pathway leads to continuously activation of PI3K/AKT and MAPK signaling and increases the abilities of cell growth, survival, and proliferation, which contributes to the occurrence of acquired resistance to pyrotinib. However, apatinib inhibits the activation of PI3K/AKT and MAPK signaling by targeting c-kit to block the downstream transduction of SCF/c-kit signaling, thereby reversing the acquired pyrotinib resistance. *P < 0.05; **P < 0.01; ***P < 0.001; ****P < 0.0001. *N.S.* not significant

To investigate whether apatinib combined with pyrotinib improves acquired pyrotinib resistance in vivo, the mice were inoculated with NCI-N87-AR cells and randomized into four groups treated daily with 10 mg/kg pyrotinib, 75 mg/kg apatinib, the combination of both inhibitors, or saline as a control (Fig. 6a). The combination group exhibited enhanced antitumor effects in NCI-N87-AR xenografts relative to those observed from monotherapies (*P* < 0.05), with no significant difference in weight change observed between the four groups (Fig. 6b–e). IHC analysis of tissues from xenografts revealed similar SCF levels in the four groups, although decreased Ki67 levels and increased cell apoptosis were observed in the combined-treatment group, with no significant difference observed in CD31 levels between the apatinib and combined-treatment groups (Fig. 6f).

## Discussion

Currently, in precision medicine, HER2-targeted therapy is widely used in the treatment of a variety of tumors. However, tumor heterogeneity and the occurrence of primary or acquired drug resistance limit the efficacy of anti-HER2 therapy alone. Therefore, a commonly accepted treatment strategy involves combining anti-HER2 therapy with other targeted drugs, resulting in enhanced therapeutic benefits due to synergy, reduced treatment-related toxicity due to lower drug doses, and decreased or no drug resistance.

The interaction between drugs is usually confirmed in vitro by isobologram analysis [19]. Since we were uncertain whether interaction of pyrotinib with apatinib in this study would be affected by tumor microenvironments, we performed in vivo analysis to allow a clinically relevant assessment. Therefore, we refer to a method derived from isobologram analysis, and use the CI to determine the presence of synergy in combined pyrotinib and apatinib treatment in vivo [20]. Our analysis revealed a CI < 1, which indicated synergy and was consistent with our hypothesis.

We initially considered the anti-vascular effect of apatinib during our investigation of the synergistic mechanism. Co-culture of apatinib-treated HUVEC cells with pyrotinib-treated HER2-positive GC cells revealed no significant difference in cell survival, proliferation, or malignant properties in the GC cells relative to controls. Because apatinib is a multi-target TKI in addition to its targeting VEGFR2, we speculated that apatinib and pyrotinib may act on tumor cells together.

Researchers have reported that the PI3K/AKT and MAPK pathways are the most important downstream signaling pathways of HER2 receptor [21]. Our data showed that HER2-positive GC cells exposed to combined pyrotinib and apatinib treatment did not exhibit significant decreases in phosphorylated levels of HER2 (p-HER2) and p-VEGFR2 relative to those observed following monotherapies, whereas p-AKT, p-mTOR and p-ERK were significantly reduced following combined treatment (Fig. 1l). Therefore, we speculated that apatinib blocked PI3K/AKT and MAPK pathways through other pathways unrelated to HER2 and VEGFR2 levels.

To investigate the mechanisms associated with pyrotinib resistance and identify potential treatment strategies, we compared the differences in pyrotinib-resistant cells and their parent cells. *KITLG* encodes a ligand for the tyrosine kinase c-kit, commonly known as stem cell factor (SCF) [22–25]. SCF binding to its receptor (c-kit) promotes phosphorylation of the PI3K regulatory subunit p85α encoded by *PIK3R1* to subsequently activate the protein kinase B (i.e., AKT1). Additionally, SCF is involved in mediating MAPK signaling. The primary function of SCF in hematopoietic cells is to enhance the effect of other growth factors, and thereby induce cell proliferation [23, 26, 27]. It also plays a similar role in promoting cell growth in solid tumors. Previous reports indicate that an SCF/c-kit autocrine loop increases cell growth via EGF family ligands by enhancing signal transduction in the PI3K and ERK pathways in breast cancer [28, 29]. Other researchers found that the activation of the SCF/c-kit pathway promoted the property of cell proliferation and invasion in colorectal and pancreatic cancers via the PI3K/AKT and/or MAPK pathways [30, 31]. In the present study, our data were consistent with these findings and demonstrated that up-regulation of SCF levels was associated with acquired pyrotinib resistance. Activation of PI3K/AKT and MAPK signaling via the SCF/c-kit interaction induced the proliferation and survival of pyrotinib-resistant cells.

Previous studies indicate that activation of alternate pathways represents an important resistance mechanism against anti-HER2 drugs [7, 32, 33]. The crosstalk between the EGFR/HER2 and SCF/c-kit pathways occurs at multiple levels, with PI3K/AKT and ERK representing crucial downstream signaling nodes. Because SCF activates PI3K/AKT and MAPK signaling through its receptor c-kit, this suggests that combining pyrotinib with c-kit inhibitors might effectively address the observed drug resistance.

The c-kit protein, which is the SCF receptor, is a member of the type III receptor tyrosine kinase family and stimulates continuous proliferation and loss of anti-apoptotic signals in tumor cells through various pathways [23]. Imatinib, a classical c-kit inhibitor, was found to be effective for combating acquired pyrotinib resistance both in NCI-N87-AR cells and xenografts. These data indicate that the SCF/c-kit signaling do play an important role in overcoming acquired pyrotinib resistance. In addition to imatinib, apatinib, which has been proven to be effective in AGC, can also effectively inhibit c-kit phosphorylation. In our study, apatinib blocked the downstream pathways of SCF/c-kit, and therefore re-sensitized pyrotinib-resistant cells to pyrotinib both in vitro and in vivo. Since apatinib also has anti-angiogenic effects, combining it with pyrotinib seems to be a better choice than that of imatinib, which could be verified by the results of in vivo immunohistochemical staining.

Interestingly, we found that the inhibitory effect of apatinib on c-kit and the downstream pathways was also observed in NCI-N87 and SNU216 cells (Supplementary Fig. 2, Fig. 1l), indicating that apatinib might show synergy with pyrotinib by targeting c-kit and inhibiting the PI3K/AKT and MAPK signaling.

The complexity of the mechanisms associated with anti-HER2 resistance suggests the existence of other mechanisms related to acquired pyrotinib resistance [34–36]. A previous study reported that application of exosomes produced by HER2-positive GC cells treated with pyrotinib promotes HUVEC proliferation, invasion, and translocation, whereas administration of apatinib counteracted these effects [37]. Although this suggested a potential mechanism of pyrotinib resistance, the hypothesis requires further confirmation both in vitro and in vivo.

This study has limitations. Although our findings are compelling, different pyrotinib-resistant cell lines need to be established, and large-scale clinical studies need to be conducted for confirmation of the results and further elucidation of the associated mechanisms. Furthermore, clinical trials are required to establish the safety and efficacy of this drug combination in vivo.

In conclusion, our study revealed that apatinib exhibited synergistic antitumor effects with pyrotinib and reversed acquired pyrotinib resistance in HER2-positive GC by targeting the SCF/c-kit/PI3K/AKT and SCF/c-kit/MAPK signaling pathways (Fig. 6g). Additionally, our findings provided evidence for the safety of the combined therapy in vivo using an animal model. These results suggest the efficacy of combined application of pyrotinib and apatinib in HER2-positive AGC patients as a novel treatment strategy that can also be applied for patients with acquired pyrotinib resistance characterized by elevated SCF expression.

## Electronic supplementary material

Below is the link to the electronic supplementary material.

Supplementary file1 (DOCX 3519 kb)

## Data Availability

All data generated or analyzed during this study are included in this published article and its supplementary information file. Further details are available from the corresponding author on reasonable request.
