# Supplementary material for: Apatinib exhibits synergistic effect with pyrotinib and reverses acquired pyrotinib resistance in HER2-positive gastric cancer via stem cell factor/c-kit signaling and its downstream pathways
Source: Gastric Cancer. 2020 Oct 8;24(2):352–67. doi: 10.1007/s10120-020-01126-9 (PMC7902570; doi:10.1007/s10120-020-01126-9)
Supplement: Supplementary file 1 — Supplementary file1 (DOCX 3519 kb) [file 10120_2020_1126_MOESM1_ESM.docx]

**Article title:** Apatinib exhibits synergistic effect with pyrotinib and reverses acquired pyrotinib resistance in HER2-positive gastric cancer via stem cell factor/c-kit signaling and its downstream pathways

**Journal name:** Gastric Cancer

**Author names:** Beibei Su, Tingting Huang, Yu Jin, Han Yin, Hong Qiu, Xianglin Yuan

**Affiliation and e-mail address of the corresponding author:** Department of Oncology, Tongji Hospital, Tongji Medical College, Huazhong University of Science and Technology, 1095 Jiefang Avenue, Wuhan, Hubei 430030, China. E-mail: [yuanxianglin@hust.edu.cn](https://wx.qq.com/cgi-bin/mmwebwx-bin/webwxcheckurl?requrl=http%3A%2F%2Fyuanxianglin%40hust.edu.cn&skey=%40crypt_d49b455c_352e0f45df6daa8b5ef5025d527c5c06&deviceid=e090834741678633&pass_ticket=tU%252FJ8LtY8DEXzzpl5O979gvzdUHvy7HbRBCU6iasX9tEA3%252Fq3c8uNTd%252B25Ze6Eux&opcode=2&scene=1&username=@2973f09bab17c14a04f4ea3d2c388939).

**The supplementary data**

**1. Supplementary Figure 1-4**

**2. Supplementary Table 1-3**

1. **Supplementary Figure**


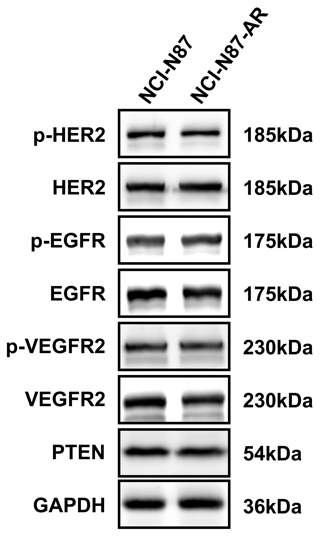


**Supplementary Fig. 1** Different protein expressions between NCI-N87 and NCI-N87-AR cells. Protein expression of HER2, EGFR, VEGFR2, PTEN and their phosphorylation status were evaluated using western blotting.


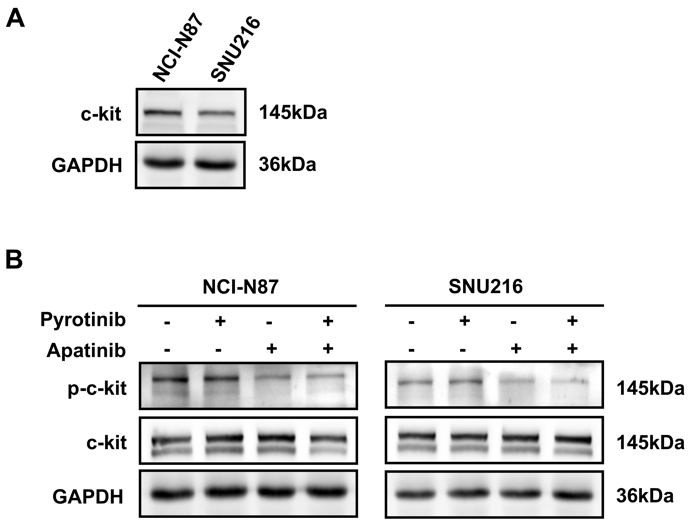


**Supplementary Fig. 2** Baseline c-kit expression and p-c-kit/c-kit expression after treatment of pyrotinib and apatinib in NCI-N87 and SNU216 cells. **A** Baseline c-kit expression in NCI-N87 and SNU216 cells was examined by western blotting. **B** NCI-N87 and SNU216 cells were treated with 0.1 μM pyrotinib, 10 μM apatinib or the combination for 24h, and then the expression of p-c-kit and c-kit was assessed by western blotting.


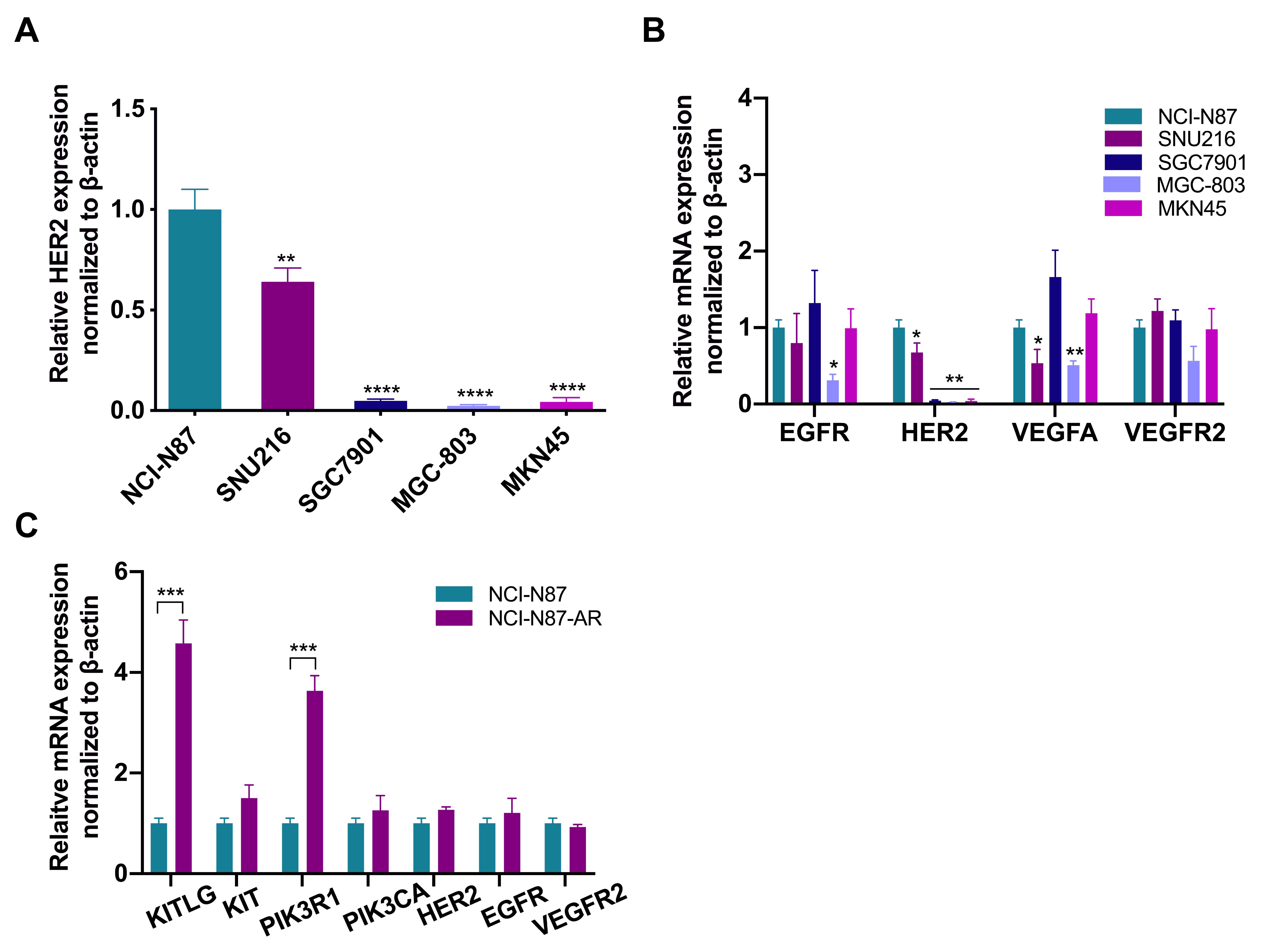


**Supplementary Fig. 3** Relative mRNA expression normalized to β-actin. **A** The mRNA levels of HER2 in five human GC cell lines. **B** The mRNA levels of EGFR, HER2, VEGFA and VGEFR2 in five human GC cell lines. **C** The mRNA levels of the indicated genes in NCI-N87 and NCI-N87-AR cells. The experiments were performed in triplicate.

**
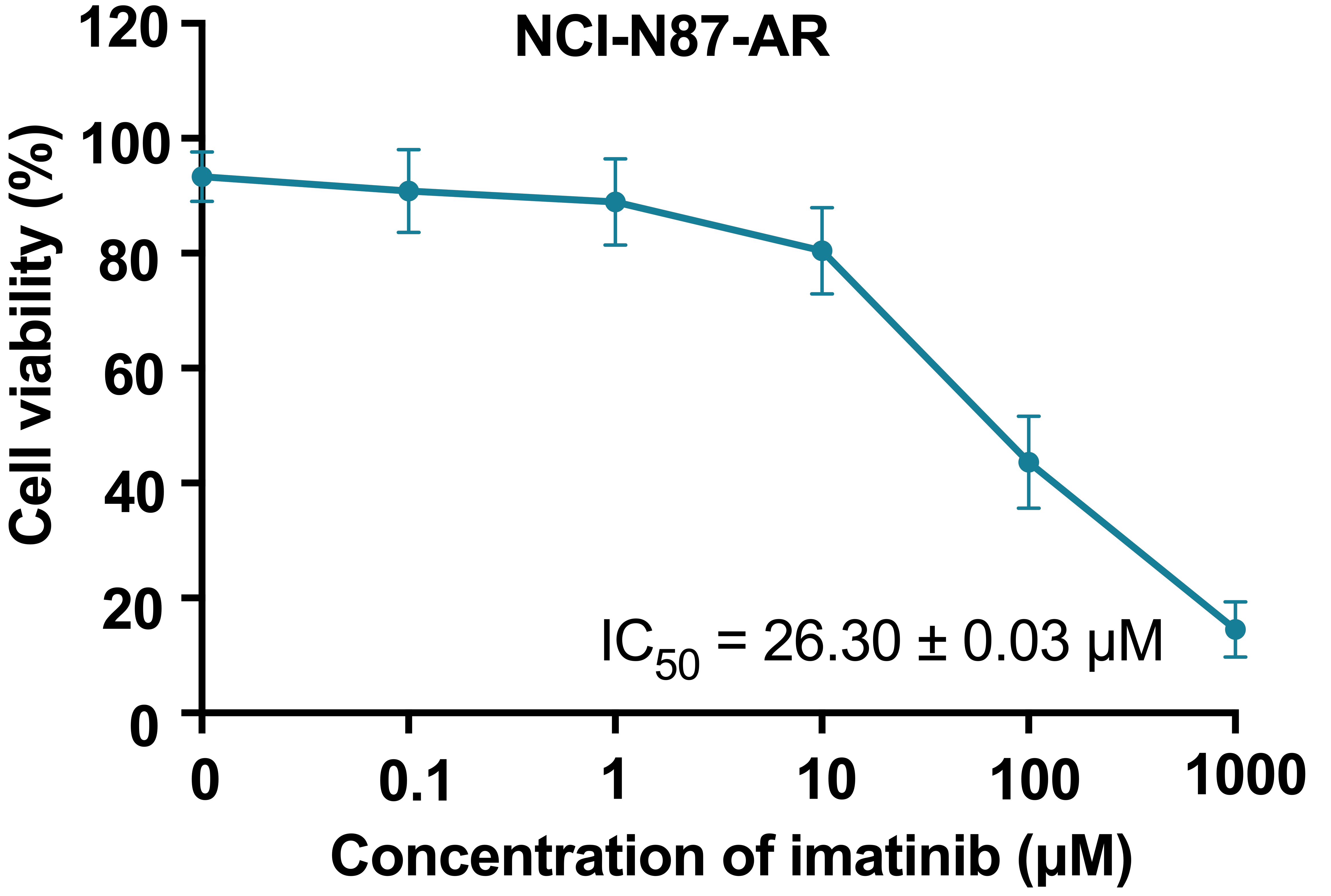
**

**Supplementary Fig. 4** The IC_50_ of imatinib in NCI-N87-AR cells. Cell viability of NCI-N87-AR cells was measured via the CCK-8 assay after different concentrations of imatinib treatments and the IC_50_ of imatinib in NCI-N87-AR cells was calculated as 26.30±0.03μM. The experiments were performed in triplicate.

1. **Supplementary Table**

**Supplementary Table 1: IC_50_ values of pyrotinib and apatinib on five gastric cancer cell lines**

| Cell Line | Tumor Type | HER2 Expression ^a^ | IC_50_ (72h) ^b^ | |
| --- | --- | --- | --- | --- |
|  |  |  | Pyrotinib (uM) | Apatinib (uM) |
| NCI-N87 | Gastric | ++++ | 0.08 ± 0.01 | 38.9 ± 1.47 |
| SNU216 | Gastric | +++ | 0.12 ± 0.02 | 33.1 ± 2.05 |
| SGC7901 | Gastric | - | ＞10 | 29.7 ± 5.45 |
| MGC-803 | Gastric | - | ＞10 | 31.4 ± 1.99 |
| MKN45 | Gastric | - | 8.35 ± 0.23 | 49.6 ± 2.44 |

a Previously reported in western blotting (Fig 1a, b).

b IC_50_ values (drug concentration required to inhibit growth by 50%) were calculated with GraphPad Prism (Version 8.0) software. Data shown are as means ± SDs of 3 independent experiments.

**Supplementary Table 2: List of primer sequences**

| Genename | Type | Sequence 5'-3' |
| --- | --- | --- |
| HER2 | Forward | TGCAGGGAAACCTGGAACTC |
|  | Reverse | ACAGGGGTGGTATTGTTCAGC |
| VEGFA | Forward | AGGGCAGAATCATCACGAAGT |
|  | Reverse | AGGGTCTCGATTGGATGGCA |
| VEGFR2 | Forward | GGCCCAATAATCAGAGTGGCA |
|  | Reverse | CCAGTGTCATTTCCGATCACTTT |
| EGFR | Forward | AGGCACGAGTAACAAGCTCAC |
|  | Reverse | ATGAGGACATAACCAGCCACC |
| β-actin | Forward | CATGTACGTTGCTATCCAGGC |
|  | Reverse | CTCCTTAATGTCACGCACGAT |
| PIK3R1 | Forward | ACCACTACCGGAATGAATCTCT |
|  | Reverse | GGGATGTGCGGGTATATTCTTC |
| KITLG | Forward | AATCCTCTCGTCAAAACTGAAGG |
|  | Reverse | CCATCTCGCTTATCCAACAATGA |
| KIT | Forward | CGTTCTGCTCCTACTGCTTCG |
|  | Reverse | CCCACGCGGACTATTAAGTCT |
| PIK3CA | Forward | CCACGACCATCATCAGGTGAA |
|  | Reverse | CCTCACGGAGGCATTCTAAAGT |
| GAPDH | Forward | AATCCCATCACCATCTTCCAG |
|  | Reverse | GAGCCCCAGCCTTCTCCAT |

**Supplementary Table 3: Detailed information of primary antibodies**

| Antibody | Supplier | Catalogue | Dilution | Host |
| --- | --- | --- | --- | --- |
| HER2 | CST | 2165 | 1:1000 | Rabbit |
| p-HER2 | CST | 2243 | 1:1000 | Rabbit |
| EGFR | CST | 4267 | 1:1000 | Rabbit |
| p-EGFR | CST | 3777 | 1:1000 | Rabbit |
| VEGFR2 | CST | 9698 | 1:1000 | Rabbit |
| p-VEGFR2 | CST | 2478 | 1:1000 | Rabbit |
| ERK1/2 | CST | 4695 | 1:1000 | Rabbit |
| p-ERK1/2 | CST | 4370 | 1:2000 | Rabbit |
| mTOR | CST | 2983 | 1:1000 | Rabbit |
| p-mTOR | CST | 5536 | 1:1000 | Rabbit |
| c-kit | CST | 3074 | 1:1000 | Rabbit |
| p-c-kit | CST | 3391 | 1:1000 | Rabbit |
| PARP | CST | 9532 | 1:1000 | Rabbit |
| Cleaved PARP | CST | 5625 | 1:1000 | Rabbit |
| Caspase-9 | CST | 9502 | 1:1000 | Rabbit |
| Cleaved caspase-9 | CST | 9505 | 1:1000 | Rabbit |
| Caspase-3 | CST | 14220 | 1:1000 | Rabbit |
| Cleaved caspase-3 | CST | 9661 | 1:1000 | Rabbit |
| SCF | Abcam | ab52603 | 1:10000 | Rabbit |
| PI3K P85α | Abcam | ab86714 | 1:1000 | Rabbit |
| p-PI3K P85α | Abcam | ab182651 | 1:1000 | Mouse |
| PTEN | CST | 9188 | 1:1000 | Rabbit |
| AKT | CST | 4685 | 1:1000 | Rabbit |
| p-AKT | CST | 4060 | 1:1000 | Rabbit |
| GAPDH | CST | 5174 | 1:1000 | Rabbit |
| α-Tubulin | CST | 2125 | 1:1000 | Rabbit |
